# Epidemiological Aspects of Crimean-Congo Hemorrhagic Fever in Western Europe: What about the Future?

**DOI:** 10.3390/microorganisms9030649

**Published:** 2021-03-21

**Authors:** Aránzazu Portillo, Ana M. Palomar, Paula Santibáñez, José A. Oteo

**Affiliations:** Infectious Diseases Department, Center of Rickettsiosis and Arthropod-Borne Diseases (CRETAV), San Pedro University Hospital-Center of Biomedical Research from La Rioja (CIBIR), Piqueras, 98, 26006 Logroño, La Rioja, Spain; aportillo@riojasalud.es (A.P.); ampalomar@riojasalud.es (A.M.P.); psantibanez@riojasalud.es (P.S.)

**Keywords:** arbovirus, tick, *Hyalomma marginatum*, *Hyalomma lusitanicum*, Crimean-Congo hemorrhagic fever virus (CCHFV), Crimean-Congo hemorrhagic fever (CCHF), Spain, western Europe

## Abstract

Crimean-Congo hemorrhagic fever virus (CCHFV) is an arthropod-borne virus (arbovirus), mainly transmitted by ticks, belonging to the genus *Orthonairovirus* (family *Nairoviridae*, order *Bunyavirales*). CCHFV causes a potentially severe, or even fatal, human disease, and it is widely distributed in Africa, Asia, eastern Europe and, more recently, in South-western Europe. Until a few years ago, no cases of Crimean-Congo hemorrhagic fever (CCHF) had been reported in western Europe, with the exception of several travel-associated cases. In 2010, the CCHFV was reported for the first time in South-western Europe when viral RNA was obtained from *Hyalomma lusitanicum* ticks collected from deer in Cáceres (Spain). Migratory birds from Africa harboring CCHFV-infected ticks and flying to Spain appear to have contributed to the establishment of the virus (genotype III, Africa-3) in this country. In addition, the recent findings in a patient and in ticks from deer and wild boar of viral sequences similar to those from eastern Europe (genotype V, Europe-1), raise the possibility of the introduction of CCHFV into Spain through the animal trade, although the arrival by bird routes cannot be ruled out (Africa-4 has been also recently detected). The seropositive rates of animals detected in regions of South-western Spain suggest an established cycle of tick-host-tick in certain areas, and the segment reassortment detected in the sequenced virus from one patient evidences a high ability to adaptation of the virus. Different ixodid tick genera can be vectors and reservoirs of the virus, although *Hyalomma* spp. are particularly relevant for its maintenance. This tick genus is common in Mediterranean region but it is currently spreading to new areas, partly due to the climate change and movement of livestock or wild animals. Although to a lesser extent, travels with our pets (and their ticks) may be also a factor to be considered. As a consequence, the virus is expanding from the Balkan region to Central Europe and, more recently, to Western Europe where different genotypes are circulating. Thus, seven human cases confirmed by molecular methods have been reported in Spain from 2016 to August 2020, three of them with a fatal outcome. A One Health approach is essential for the surveillance of fauna and vector populations to assess the risk for humans and animals. We discuss the risk of CCHFV causing epidemic outbreaks in Western Europe.

## 1. Introduction

Crimean Congo Hemorrhagic Fever (CCHF) is the most widely distributed tick-borne viral disease in the world (Africa, Asia, eastern and South-eastern Europe and recently, South-western Europe), and the second one (after dengue) among viral hemorrhagic fevers [1,2,3,4]. The clinical course of the infection varies from asymptomatic to severe and even fatal cases (3–40%) [5,6]. In fact, asymptomatic infections seem common [7], reaching up to 90% cases in some studies from hyperendemic areas [8]. CCHF is transmitted to people by the bites of hard ticks (or crushing of engorged specimens) and/or by direct contact with secretions, fluids or tissues of viraemic animals (slaughtering activity, animal abortion, farmers, animal husbandry, etc.) or with infected humans (blood, secretions and other biological fluids) without protective measures [9,10]. In the sanitary environment, nosocomial outbreaks are well reported [11], including those related to aerosol generation [12]. Nevertheless, the risk of suffering CCHF after exposition to infected biological fluids seems to be lower than the risk of healthcare-related exposure related to other hemorrhagic viruses such as Ebola [13]. There is an increased risk of nosocomial transmission when the disease is not suspected or the prevention measures are not used [14]. Nosocomial transmission of CCHF seems to be common in pregnancy [15]. Vertical transmission has been also reported [16,17], and sexual contact may represent an additional risk of CCHF transmission [18,19]. Laboratory-acquired accidental cases when handling viral material has been also described [9].

The etiological agent is an enveloped, segmented, negative-sense, single-stranded (ss) RNA arbovirus named Crimean-Congo hemorrhagic fever virus (CCHFV). It belongs to the genus *Orthonairovirus* (family *Nairoviridae*, order *Bunyavirales*), within the realm *Riboviria* (a new megataxonomic taxon rank), according to the most recent classification based on the advances in viral genomic and metagenomic comparison analysis [20,21]. The viral genome consists of three RNA segments: small (S), medium (M) and large (L), which encode the viral nucleoprotein (NP), the glycoprotein precursor (GPC) that yields the structural glycoproteins (G_N_ and G_C_), and the RNA-dependent RNA polymerase, respectively [22]. CCHFV exhibits higher genetic diversity than other tick-borne viruses, which reveals a wide dispersion of the virus [23]. Since increasing complete genomic sequences of CCHFV are becoming available, new viral classifications are appearing in the last years. Previously classified into six main geographical clades [6], up to nine genetically different clades are currently proposed for CCHFV, according to the phylogenetic analysis of the complete genetic sequence of the S RNA segment of the genome, and based on the geographical origin [24]. Four of them are predominantly distributed in Africa, two in Asia and three in Europe, as follows: Africa-1 (genotype I), Africa-2 (genotype II), Africa-3, (genotype IIIa) and Africa 4 (genotype IIIb); Asia-1 (genotype IVa) and Asia-2 (genotype IVb); Europe-1 (genotype V), Europe-2 (genotype VI) and Europe-3 (genotype VII) [24].

**Figure 1:**
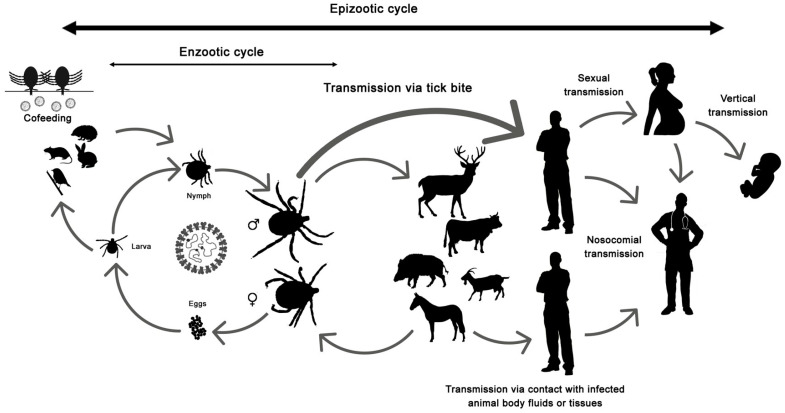
Crimean-Congo hemorrhagic fever virus (CCHFV) cycle and its routes of transmission.

The disease only affects humans, but CCHFV lives in a natural cycle affecting wild mammals, livestock, birds and ticks. Hard ticks are reservoirs of the virus in the nature through transovarial, transstadial and venereal transmission [25]. The cycle of CCHFV is shown in Figure 1.

The virus has been detected in at least 35 (32 hard and three soft) tick species in the world, although only *Hyalomma* ticks are recognized vectors, and specifically *Hyalomma marginatum* (widely distributed in the Mediterranean area) seems to be the most efficient vector [26]. In fact, the distribution of *Hyalomma* ticks overlaps with the distribution of CCHF cases [2,9,26,27]. Apart from *Hyalomma* spp., *Rhipicephalus bursa* seems also relevant for CCHFV transmission [26]. Despite CCHFV has been detected in eggs of *Dermacentor marginatus*, there are no studies about the ability of *D. marginatus* for the transmission or maintenance of the virus [26]. The prevalence of CCHFV infection in ticks is variable in different tick species. Studies about prevalence of CCHFV in ticks from western Europe are included in Table 1. 

While birds can disseminate the agent by carrying infected ticks (immature specimens) [27,41], most avian species appear refractory to infection based on numerous studies in which viremia or serological evidence of infection could not be demonstrated after their experimental inoculation with CCHFV [9]. Mammals and ostriches are amplifiers of the infection. The potential role of reptiles in the maintenance of CCHFV in nature has been suggested after detection of the virus in tortoises as well as in their feeding ticks (*Hyalomma aegyptium*) and in unfed *H. aegyptium* in the field [42].

CCHFV has been classified as a biosafety level 4 pathogen. This means that it has high risk of aerosol-transmission and causes frequently fatal infections. Although treatment with ribavirin has shown satisfactory results, its use is controversial [43]. In addition, no commercial vaccines are available to date in the EU member States. Nevertheless, an inactivated suckling mouse brain vaccine started to be used in 1974 in Bulgaria for the prophylaxis of boarder army units, medical workers, agricultural workers and other people living in endemic regions [44]. Inactivated vaccines based on cell-culture seem more protective than mouse brain-derived vaccine preparations tested in animal models, and the search for new candidates using immune-informatics looks promising [45,46]. It is also a category C bioterrorism and/or biological warfare agent since it could be produced and massively disseminated [47]. CCHF has appeared in the WHO list of the most important emerging infectious diseases likely to cause major epidemics since 2015 and it is considered at present as a priority disease with pandemic potential [48]. The current pandemic situation caused by SARS-CoV-2 has evidenced, even more, that surveillance, prevention and control of zoonotic viral disease threads are critical for global survival. This fact, joined to the recent detection of CCHFV in ticks in South-western Europe and the subsequent emergence and spread of CCHF human cases in nearby areas, justify this review. Our aim is to summarize the current knowledge of distribution of CCHF in South-western Europe as well as to assess the risk surveillance in order to contribute to the awareness and preparedness against this zoonosis for the near future. Up to February 2021, original research manuscripts, reviews and opinion articles about CCHFV, tick species, reservoirs and human cases in western European countries were searched in PubMed, Google Scholar, Science Direct, Scopus and Web of science using the following terms: “Crimean-Congo hemorrhagic fever”, “CCHF” or “CCHFV”, filtered by the possibilities of “ticks”, “tick species”, “tick distribution”, “migratory birds”, “Europe”, “imported” “travel”, “the name of the country”, “imported livestock”, “nosocomial”, “transmission”, “vertical”, “sexual”, “aerosols”, “classification”, “genotypes”, “phylogeny”. Articles used for this literature review were further selected based on their title, abstract and content (if available).

## 2. A Touch of History about CCHF: The Experience in Spain

There are complete reviews that discuss the historical perspective of CCHF from the first description of a hemorrhagic syndrome related to an arthropod-bite in Tajikistan in the XII^th^ century to the modern era, when the same virus was isolated from patients in Crimea and Congo [2,9]. CCHF is endemic in Africa, Asia, in the Balkans as well as in many countries from eastern Europe and the Middle East, and the situation of epidemics has been reported in countries from the East Mediterranean coast for the last two decades [6,49,50]. In the South-West of Europe (Iberian Peninsula), the first finding about the presence and circulation of CCHFV dates back to 2010, when the virus was detected in *Hyalomma lusitanicum* ticks collected from deer in Cáceres [31], in the framework of a project to investigate the potential risks of establishment of CCHFV and tick-borne encephalitis virus (TBEV) in Spain (project no. PS09/02492, Ministry of Economy and Competitiveness, Spain). Previously, there had been only indirect evidence of the virus based on antibody detection in two out of 258 human sera from southern Portugal [51]. With the exception of some travel-associated CCHF cases in Germany, France and UK as well as limited infections due to their derived nosocomial transmission [44,52,53,54,55,56,57,58,59,60] (Table 2), no autochthonous cases of CCHF had been reported in western Europe until the first two cases (one of them, fatal) in Spain in 2016 [61].

In the Iberian Peninsula, *H. marginatum* and other *Hyalomma* species (*H. lusitanicum*) were known to be present in the South of Portugal and in the South-West of Spain before year 2000 [62,63]. But at that time it was very strange to observe human tick-bites by *Hyalomma* spp. However, a growing number of people bitten by *H. marginatum* who came to our hospital was noticed in the ensuing years [64,65,66], and the same finding was reported in other areas from Spain such as Castilla-León, where *H. marginatum* went from the fourth to the second most anthropophilic species among ticks biting people from 1997 to 2007 [67,68]. It was known that *H. marginatum* was the vector of a *Rickettsia* species, *Rickettsia aeschlimannii*, responsible for a boutonneuse fever-like that was circulating in the Mediterranean area and in Africa [64,69], and it was also the vector of an exotic disease for us, the CCHF, non-previously described neither in Spain nor in the surrounding countries.

With this background, our group in collaboration with the Prof. Estrada-Peña (University of Zaragoza) had the opportunity of screening CCHFV in *H. lusitanicum* specimens, collected over deer in Cáceres (South-West of Spain), on the edge of the Tajo river near the Portuguese border. A total of 117 specimens were screened by PCR targeting the small segment of CCHFV, and the virus was detected for the first time in South-western Europe in 2010 [31]. This finding triggered a number of questions:

(1) ‘Was the virus circulating silently in Spain?’ This seemed unlikely. The disease usually presents as a subclinical infection but it may lead to hemorrhagic complications and be highly lethal, and no human cases had been previously diagnosed in the country.
(2) ‘How had the CCHFV arrived to Spain?’ The virus could have been introduced into Spain by the transport and trade of live animals harboring infected ticks. There are several instances of serological evidence of CCHFV-*Hyalomma*-infested and CCHFV-infected imported livestock (antibodies) from endemic to non-endemic countries, such as camels from Sudan and Kenya imported into Egypt [70], sheep, goats and cattle imported into Saudi Arabia from Sudan [71], or livestock imported into Oman from Somalia [72]. Seroprevalence studies in livestock had shown a high prevalence of exposure in endemic countries [73]. However, as long as the cattle is deparasitized, this route did not seem important for viral dispersal. Parasitized cattle could contribute to amplify the pathogen, although the viremia is brief (7 days in experimental animals) [74]. At that time, no studies had been performed either in Spain or in western European countries in cattle, with the exception of a serological survey for CCHFV antibodies in 141 goat sera from Portugal without evidence of the virus [51]. On the contrary, there had been indirect evidences of CCHFV based on the detection of antibodies in blood sera from two out of 19 bats in France, from an area bordering with Spain [27].
(3) ‘Were there other sources for virus release?’ From our first report describing CCHFV in Spain [31], we hypothesized about the introduction of the virus through migrating birds from Africa that carried infected ticks because CCHFV amplicons detected in Spanish *H. lusitanicum* showed the highest similarity with those from Mauritania and Senegal (genotype III), and were phylogenetically far from the European ones (up to that moment, genotypes V and VI) [31]. In addition, we had previously confirmed the spread of ticks and tick-borne bacteria (*Anaplasma, Borrelia* and *Rickettsia*) by birds [75]. Based on these findings, in 2011, thanks to a collaboration with bird ringers from the Aranzadi Society, we had the opportunity to study ticks collected from 546 different migratory birds from Zouala (Morocco). A total of 21 bird specimens from five species (*Phoenicurus phoenicurus, Erythropygia galactotes, Iduna opaca, Acrocephalus scirpaceus* and *Iduna pallida*) were parasitized with 52 *H. marginatum* ticks (larvae and nymphs). Four out of six pools were positive for CCHFV and the obtained sequences were identical to Sudan AB1-2009 and Mauritania ArD39554 CCHFV strains, and nearly 99% similar to those previously amplified in Spain [41]. Thus, our hypothesis was confirmed with the detection of infected ticks from migratory birds that could arrive into the Iberian Peninsula from Africa. We had changed the CCHFV distribution map with the findings of the virus in Spain and Morocco. The role of birds as transporters of the CCHFV was reinforced with the finding of the Africa 3 viral genotype in ticks from migratory birds collected in a Greek island [76].

With all these data, in October 2011, the Health Alert and Emergency Coordination Center (CCAES) from the Ministry of Health, Social Policy and Equality, supported by a group of experts, published the first report on the situation of CCHFV in Spain [77]. It was then estimated that there was a low risk for human infections in our country, although a multidisciplinary approach of surveillance and control of the virus was recommended. Again with financial support of the Ministry of Economy and Competitiveness (Spain) (project no. PI12/02579), we continued to investigate *H. marginatum* specimens collected from humans and birds in the North of Spain (years 2009–2015). A total of 12 adult ticks from humans and 149 immatures (125 from La Rioja and 24 from Palencia) from 52 birds (21 bird species) were screened for CCHFV. No virus was detected in *H. marginatum* neither from humans nor from birds, although the risk of CCHFV-infected ticks occurred in our country [32]. Subsequently, under the framework of the same project, and thanks to an interdisciplinary collaboration, we also searched for the presence of CCHFV in 2053 adult ticks (1333 *H.* marginatum, 680 *H. lusitanicum* and 40 *R. bursa*) from vegetation, cattle and sheep collected in Extremadura (near the area of the first detection in Spain) and in other provinces from Spain (years 2013–2015), with negative PCR results [33].

From 2011–2013, members from the Arbovirus and Imported Viral Diseases Laboratory from the Spanish National Center of Microbiology (Madrid, Spain) also screened 681 ticks (*Rhipicephalus* spp., *H. lusitanicum* and *H. marginatum*) for the presence of CCHFV, and the detection of 24 *H. lusitanicum* infected with CCHFV collected from deer in Extremadura corroborated our previous finding. From a total of 272 more ticks analyzed in 2014, nucleotide sequences were inconclusive in three positive samples collected over deer, red fox and cattle [78].

Once the virus had been shown to be present in Cáceres area, in 2014 we aimed to assess the risk of exposure to CCHFV in humans from rural areas (hunters, livestock farmers…) near the virus detection point as well as in people at risk of tick-bites (foresters, forest rangers…) and/or diagnosed of a tick-borne disease from La Rioja. A total of 228 serum samples were screened by indirect immunofluorescence assay (IFA) and none of them showed IgG antibodies against CCHFV [33]. These data, joined to the previous studies carried out by our team in northern Spain [32,66], suggested that the virus was not widely distributed in Spain and the risk of infection acquisition was low at that time.

A recently published surveillance study on 1579 ticks from wildlife, domestic animals and vegetation in four Spanish regions (years 2011–2015) found a CCHFV infection rate of 2.78% in *H. lusitanicum* collected from deer and cattle in Cáceres, a percentage similar to those found in endemic European countries. Only 1/238 *H. marginatum* removed from a cow yielded positive CCHFV results. Analyzed viral sequences belonged to the Africa-3 clade (genotype III), and showed the occurrence of four variants that diverged to produce two lineages. Any of these possibilities could explain the phylogenetic analysis: ‘Was there a common ancestor?’ or, on the contrary, ‘did different viruses arrive to Spain by birds and/or by international cattle trade?’ [36]. This study helped unravel one of our initial questions: ‘Was the virus circulating silently in Spain?’ The intermittent activity of the CCHFV detected in Cáceres supported the idea of a cryptic circulation of the virus that, as a result of different factors, may periodically originate outbreaks [36,43]. In addition, the detection of antibodies to CCHFV in two human sera samples from Portugal in the 80s suggested that the virus had been introduced into the Iberian Peninsula approximately 50 years ago [51], as corroborated with recent phylogeny and molecular clock estimations [34].

### **A Touch of History about CCHF: The First Autochthonous CCHF Cases in Western Europe** 

In 29 August 2016, the alarm was activated with the admission to an ICU of a nurse that had been exposed to the blood of a patient who had recently died due to multiple organ failure. The index patient (without any history of travel) had been probably bitten by a tick in a village in Ávila (North East of Cáceres) a few days before. Based on these facts, we were consulted and strongly recommended ruling out a CCHF. The disease was confirmed in both cases. More than 400 people with previous contact with both patients were under epidemiological surveillance during 14 days and no one was ill. The lack of seropositivity (no IgG antibodies against CCHFV) in the analyzed serum samples from 49 people highly exposed to these two cases in 2016 (most of them from the sanitary environment) corroborated that measures for prevention and control of infection are effective and must not be never relaxed [79].

The map of CCHF cases had definitively changed in Europe in 2016, and it included Spain. The fatal case was published contributing to a better understanding of the disease since the patient had a necropsy [80]. From an epidemiological perspective, it is worth noting that the viral sequences detected in *H. lusitanicum* ticks by our team in 2010 were similar to those found in plasma samples from the two patients six years later, although probably the tick-bite of the index patient occurred 200 km far away (Africa-3 clade, genotype III). The complete genomic sequence of the virus from the patient 2 (secondary case) supported these data [31,80,81]. The complete S, M and L segment sequences of CCHFV infecting one semi-engorged female *H. lusitanicum* out of 210 ticks (45 pools) collected from deer in Cáceres in 2014 were obtained by RT-PCR, and again corroborated our results, although the virus could not be isolated [34].

As a consequence of these human cases, in September 2016, an updated report about the status and risk assessment of CCHF transmission was recorded in Spain. Experts considered that the risk remained low [78]. The following report (April 2017) provided data on the prevalence of ticks’ infection removed from livestock and wild animals in regions near the Cáceres area where we had previously found the virus. More than 9500 ticks were studied in 3959 pools (September 2016–February 2017). None of the tick pools from domestic livestock tested positive for CCHFV, whereas 128 pools from wild animals collected in seven out of 11 livestock areas (four in Extremadura; one in Madrid; one in Castilla-La Mancha and one in Castilla-León) were positive. Most of them belonged to *H. lusitanicum* removed from deer. After notification of the first autochthonous case and having demonstrated the circulation of the CCHFV in new areas, the risk in Spain was still considered low, although more sporadic cases could appear [35]. We still thought that ‘what is not sought is not found’ and that we had to remain alert with the CCHF [82].

As a result of a CCHFV-screening carried out in 1356 ticks (452 pools) collected from vegetation (80% *H. lusitanicum*) in Andalucía, Castilla-La Mancha, Castilla-León and Madrid (May–October 2017), seven pool ticks from Andalucía (5/37 pools in Cádiz and 2/22 pools in Córdoba) were positive. These data are still unpublished, although they were notified by the Ministry of Health in 2019 [38].

A serosurvey in 516 asymptomatic adults (blood donors) from western Spain (Castilla y León) was performed from May 2017 to May 2018 using four assays (immunoenzymatic and IFA). Seroprevalence values (IgG antibodies) were 0.58–1.16%, according to the positive results for at least two or one of the assays, respectively. No IgM-positive results were found [83].

According to the partial sequence of the S segment, the circulation of CCHFV genotypes different from the previous one detected in Spain (Africa-3) has been recently documented in 129 positive ticks [37]. When 613 ticks from hunter harvested wild ungulates in South-western Spain in September-December 2017 were screened for CCHFV, different sequences within the genotype Europe V (commonly designated Europe-1) were detected in *H. lusitanicum* from deer and wild boar and in *D. marginatus* from wild boar (sequences similar to human isolates from Russia and Iran), probably introduced from eastern Europe. In addition, genotype Africa IV (designated genotype IIIb, Africa-4, according to Gruber et al. [24]) was found in one *H. lusitanicum* from a deer (similar to an isolate from Nigeria), probably coming from Africa through migratory birds, as previously suggested [31,41]. Curiously, in more than one case, co-feeding CCHFV-infected ticks collected from the same ungulate host exhibited different genotypes [37].

After notification of the third CCHF case in 2018 (a hunter who stated to have been bitten by a tick in Badajoz), a fourth retrospectively diagnosed case of a patient discharged in 2018, and of which some epidemiological data were unknown (he had an eschar and meant to have been in contact with livestock), was added to the list in a new updated report (July 2019) about the situation and evaluation of the transmission risk of CCHFV in Spain [38].

On the one hand, details about the genetic characteristics of the viral sequence corresponding to the patient 3 (Badajoz, 2018) showed a reassortment event. The M and L segments were within genotype III (Africa-3), while S segment corresponded to a different genotype [84]. On the other hand, data for the fourth patient (Salamanca, 2018) have been very recently completed within the framework of a cross-sectional study of 133 patients who presented acute undifferentiated febrile syndrome (May–October 2017 and 2018) at the University of Salamanca Hospital [85], and were screened for CCHFV using molecular and serological assays. Patient 4 was the only positive case confirmed by molecular assays. Genetic analysis of the viral sequence of this patient, based on the partial S segment and also on short fragments of M and L segments, showed that it corresponded to genotype V (98% similarity with a Russian strain of European origin). This was the first time that genotype V was detected in humans in Spain, although it had been previously detected in ticks from wildlife in South-western Spain [37]. Moreover, three more cases (patients 8–10) showed positive IgG serological results by two Enzyme-Linked Immuno-Sorbent Assay (ELISA) and one of them showed undetermined results but no one was confirmed by molecular methods [85]. Data regarding human cases diagnosed by molecular tools in Spain (the only ones reported in western Europe up to date) are summarized in Table 3. The studies in which serological evidence of human infection has been reported from western Europe are showed in Table 4. Seroprevalence studies of CCHFV-infected livestock in western Europe are listed in Table 5.

The report by Sierra et al. also contained unpublished data about serological studies in animals [38]. A total of 639 wild animals and 1102 domestic animals captured in 2018 in Extremadura, Castilla-La Mancha, Castilla-León, Madrid and Andalucía had been screened for viral antibodies using ELISA ID Screen CCHF double-antigen multi-species. Seropositivity had been detected in all regions, finding 2–79% seropositive wild animals and 4–16% domestic ones. Detailed information about the percentages found in wild and domestic animals according to the zones is showed in Table 5. In addition, in animal samples from Ávila collected in 2016 (the place and year of the infected tick associated to the index patient), CCHFV seropositivity was 58% (*n* = 67) in wild animals and 33% (*n* = 79) in domestic ones. Unfortunately, no confirmatory assays could be performed [38]. The known presence of established *Hyalomma* populations in certain zones of the country, the circulation of CCHFV in ticks and animals in areas near the first virus detection area, and the notification of several sporadic human cases led to reconsider the risk in Spain from low to moderate at that time. Nevertheless, the impact of the illness was still considered low in 2019 [38].

A cluster of three more confirmed cases (patients 5–7) within a short period of time (from June to August 2020) and in a limited geographic region (the South of Salamanca province) justified a new rapid risk evaluation report [86]. They corresponded to three men (one of them with a fatal outcome) who had been bitten by a tick and lived in areas close to those where the virus and the vector had been previously found. It is worth mentioning that one seropositive blood donor from the serosurvey study previously conducted in Castilla y León was also from the ‘hot’ region (the South of Salamanca province) [83].

As a result of the three communicated cases in Salamanca in the summer of 2020, a new investigation focused on the vectors, animals and people was immediately started in the area. A total of 121 ticks from animals (including 11 *H. marginatum* from cattle) and vegetation were found, and the study is still ongoing.

In western Europe, the studies about the presence of antibodies to CCHFV in livestock outside the Iberian Peninsula is limited to a serologic survey in cattle, sheep and goats (*n* = 3890 animals) from Corsica (years 2014–2016) that has showed 13.3% of seropositive cattle (231/1731), 3.1% of goats (32/1035) and 2.5% of sheep (28/1124) [87] (Table 5).

## 3. What Is the Potential Future Impact of CCHF in Western Europe?

The recent emergence of CCHF in South-western Europe could lead to sporadic case outbreaks being endemic in this region (Spain) or become an epidemic (spreading to new areas). The Greek experience shows the most favorable situation. CCHFV was detected in 1975 in *R. bursa* ticks from Greek goats but no human cases were reported from this country until 2008 [88,89]. Previously to the confirmation of this autochthonous case, antibodies against the virus were detected in humans [90,91]. Nevertheless, up to date, only one more case has been reported from Greece and, it was imported from Bulgaria [92]. In contrast, the first CCHF human case was reported from Turkey in 2002, and 15 years later more than 11,000 cases had been notified, being endemic in many areas with a fatality rate close to 5% [42,93].

To predict the course of the disease in Spain, the sole western European country with autochthonous confirmed cases, or the incursion in neighboring countries is challenging. As it has been displayed in this review, there are several factors involved in the epidemiology of the CCHF, and presumably a combination of biological and environmental factors is responsible for its incidence [2,94]. The presence of the CCHFV virulent strains, competent vectors, reservoirs (the tick) and amplifiers (vertebrate hosts) of the virus are essential for the emergence of the disease, but a suitable environment is needed. The introduction of immature developmental stages of *H. marginatum* into western Europe through bird migrations has been reported [95,96,97,98] but local permanent *Hyalomma* have been only recognized in Portugal, Spain and France [32,96,99]. Tick-infested and/or infected (viraemic) wildlife or livestock to western European countries has been well documented, and even infected ticks [100]. Limited findings of adult *H. marginatum* and/or *Hyalomma rufipes* (formerly considered a subspecies of *H. marginatum* and also vector of CCHFV), most of them attached to horses and all showing negative results for CCHFV, have been recorded in Germany [30,101,102,103], UK [39,40], Austria [28] and The Netherlands [104]. In these cases, non-native immature *Hyalomma* ticks probably linked to the arrival of migrating birds were transmitted to the local fauna and molted into adults due to favorable conditions (warmer and dryer weather) outside the known distribution areas. Nevertheless, the potential introduction of non-autochthonous adult ticks associated to human travel cannot always be ruled out [101] and it has to also be considered that animals can travel with ticks and become the via of introduction of exotic tick species in new areas, as it occurred with one *H. lusitanicum* imported into the UK on a dog that had recently returned from Portugal [105]. In addition, other competent vectors, such as *R. bursa,* broadly distributed in the Mediterranean region [26,106], should be considered.

For the emergence of outbreaks, the tick–vertebrate–tick enzootic cycle of the virus should be established (and data in Spain points in that direction), and the exposition of susceptible people to the infected competent vector and/or to the virus (virulent strains) would be also necessary (the risk is higher in people from rural areas and/or with professions at risk). To date, the ‘hot spot’ of CCHFV infection seems to be more related to a certain area (Central-West and South-western Spain) and, hopefully, more closely associated to wild animals, but the ‘spill-over’ into livestock is a fact, and the demonstrated capacity of the virus for adaptation, among other factors, makes it a dangerous threat in our environment. CCHF, as other vector-borne viral diseases, is affected by dynamic factors such as globalization, climate change, social and cultural changes, alterations of land uses, habitats fragmentation, loss of biodiversity, introduction of exotic species, etc. [107,108,109,110,111,112]. Whether the vector in Spain is *H. marginatum* or *H. lusitanicum* is still not clear. *Hyalomma* ticks are abundant in southern Europe and a trend of their increasing human tick-bites has been observed for more than a decade. Thus, according to our data, the number of *Hyalomma* spp. detached from humans at San Pedro University Hospital (La Rioja, Spain) has almost doubled if we compare the years 2009–2014 with 2015–2020 (data not shown). All these facts, among others, combined with the different genotypes detected in ticks and humans in different areas from Spain suggest the potential establishment of a CCHFV transmission cycle. Therefore, a greater awareness and surveillance of this threat is needed. Strategies based on a One Health approach are essential for the prevention, contention, and control of the CCHF in western Europe [113]. To improve detection, diagnosis, treatment and prevention approaches under a global and coordinated perspective is essential.

**Addendum**: After submission of this review, an early release article (not the final version) about the CCHFV genetic sequence corresponding to the fatal case of patient 3 (Badajoz, 2018) from Spain was published online [https://wwwnc.cdc.gov/eid/article/27/4/20-3462_article (released on: 9 March 2021)]. Based on these data, the reassortant S segment was similar to genotype IV and related to a Nigeria strain that differed from the Asia strains of this genotype. Other authors [24] had already included this same S sequence from Spain within a new genotype designated IIIb (Africa-4). To reach a consensus about designation of genotypes, genetic linages and groups is a pending challenge.

## Figures and Tables

**Table 1:** Prevalence of Crimean-Congo hemorrhagic fever virus (CCHFV) in ticks.

Country	Dates	% of Infection Rate (No. of Positive Samples/No. of Analyzed Ticks) ^1^		Source	Reference
		PI	MIR (No. of Pools)		
Austria	2018	0 (0/1) *H. marginatum*		Migratory bird (presumably)	[28]
Corsica (French island)	2014–2015		0 (0/1015) (332 pools) 0 (0/362) *H. marginatum* (89 pools) 0 (0/518) *R. bursa* (108 pools) 0 (0/135) *H. scupense* (135 pools)	Cattle, goat, sheep, horses, dogs, wild boards, mouflons	[29]
Germany	2015	0 (0/1) *H. rufipes*		Horse	[30]
Spain	2010		1.7 (2/117) *H. lusitanicum* (12 pools)	Deer	[31]
	2009–2015	0 (0/161) *H. marginatum*		Asymptomatic patients, birds	[32]
	2013–2015		0 (0/2053) (229 pools) 0 (0/1333) *H. marginatum* (151 pools) 0 (0/680) *H. lusitanicum* (74 pools) 0 (0/40) *R. bursa* (4 pools)	Vegetation, cattle, sheep	[33]
	2014–2015		0.5 (1/208) (45 pools) 0.5 (1/204) *H. lusitanicum* (NA) 0 (0/2) *Dermacentor* spp. (NA) 0 (0/4) *Rhipicephalus* spp. (NA)	Deer	[34]
	2016–2017		1.35 (128/>9500) (3959 pools) NA *H. lusitanicum* (NA) NA *D. marginatus* (NA) NA *Rhipicephalus* sp. (NA)	Wild or domestic animals ^2^	[35]
	2011–2015	2.78 (44/1579) 4.0 (43/1079) *H. lusitanicum * 0.4 (1/238) *H. marginatum* 0 (0/46) *Rhipicephalus* spp. 0 (0/3) *I. ricinus * 0 (0/1) *Dermacentor* sp. 0 (0/212) not identified		Vegetation, deer, fallow deer, red fox, cattle, sheep, wild board ^3^	[36]
	2017	21.0 (129/613) 20.2 (119/589) *H. lusitanicum* 41.7 (10/24) *D. marginatus*		Red deer, wild boar, fallow deer, roe deer	[37]
	2017		0.5 (7/1356) (452 pools) ^4^	Vegetation	[38]
UK	2018	0 (0/1) *H. rufipes*		Horse	[39]
	2018	0 (0/1) *H. marginatum*		Vegetation ^5^	[40]

*H. marginatum: Hyalomma marginatum; R. bursa: Rhipicephalus bursa; H. scupense: Hyalomma scupense; H. rufipes: Hyalomma rufipes; H. lusitanicum: Hyalomma lusitanicum; D. marginatus: Dermacentor marginatus; I. ricinus: Ixodes ricinus*; ^1^ Total infection rate [PI: Prevalence of infection (data from individual ticks), MIR: Minimum infectious rate (data from pools)], and corresponding of each tick species analyzed; ^2^ All positive samples corresponded to ticks collected from wildlife, mainly H. lusitanicum from deer; ^3^ Positive samples were collected from deer (*n* = 41) and cattle (*n* = 3); ^4^ The majority of them (80%) corresponded to H. lusitanicum; ^5^ The tick was crawling on the leg of a man; NA: Not available.

**Table 2:** Travel-associated Crimean-Congo hemorrhagic fever (CCHF) cases in western Europe.

Year	Country of Infection	Country of Importation	Transmission Route	Age (Years)/Gender	Diagnosis	Occupation/Reason for Travel	Secondary Infection	Outcome	Reference
1997	Zimbabwe	UK	Unknown	78 F	Serology	Leisure	None	Fatal	[53]
2001	Bulgaria	Germany	Unknown	NA	Unknown	Leisure	NA	Survivor	[44]
2004	Senegal	France	Unknown	60 F	Serology & PCR	Business (voluntary radiology technician)	None	Survivor	[54]
2004	Senegal	France	Possible tick bites	72 F	Serology & PCR	Leisure	None	Fatal	[55,56]
2009	Afghanistan	Germany	Frequent outdoor activities, tick bites, and exposure to undercooked goat meat and blood	22 M	Serology & PCR	Soldier (US)	Nosocomial transmission to 2 people: Both survived	Fatal	[60]
2012	Afghanistan	UK	Animal slaughtering, contact with blood and other tissues of infected animal	38 M	PCR	Leisure	None	Fatal	[57,58]
2014	Bulgaria	UK	Tick bite and tick crushing	70 M	Serology & PCR	Leisure	None	Survivor	[59]

NA: Not available.

**Table 3:** Cases of Crimean-Congo hemorrhagic fever (CCHF) in Spain (confirmed by molecular biology tools).

Patient No.	Age	Gender	Symptoms Onset Date	Area (Province)	Tick-Bite	Risk Activity	Clinical Signs	Clinical Suspicion	Initial Treatment	CCHF Diagnosis Confirmation	Outcome	Secondary Cases	Reference
1	62	M	16/08/2016	San Juan del Molinillo (Ávila)	Not confirmed (he noticed a tick on his left knee)	Walk in countryside	2-day history of high fever, abdominal pain, malaise, nausea, and diarrhea. Next day: Severe coagulopathy, with macroscopic hematuria, purpuric skin lesions and hematomas, a low platelet count, and prolonged prothrombin and partial thromboplastin times. On the seventh day of illness: macroscopic hematuria, worsening of purpuric skin lesions and hematomas, fulminant hepatic failure, severe respiratory insufficiency, encephalopathy, hypoglycemia, and severe metabolic acidosis. 24 h later: distributive shock, oliguric renal failure, very high liver-enzyme levels, and persistent metabolic acidosis	NA	DOX	01/09/16 (post- mortem) PCR (+) IgG (−) IgM (−)	Fatal (24/8/16)	Yes, patient 2	[80]
2	50	F	27/08/2016	Madrid (Madrid)	No (nosocomial transmission)	ICU nurse of patient 1 (19–23 august)	First day: Asthenia, and arthromyalgias. On the second day: presence of petechiae, thrombocytopenia, and a mild increase in aminotransferase levels. On the third day of illness, vaginal bleeding started, coinciding with expected time of her normal menstruation period.	CCHF (4th day)	RBV (1000 mg every 6 h and reduced to 500 mg)	28/08/16- 15/09/16, PCR (+) IgG (+) IgM (+) Virus isolation	Survivor	No	[80,81]
3	74	M	04/08/2018	Helechosa de los Montes (Badajoz)	Not confirmed (suspicion of tick bites)	Hunting	Fever, abdominal pain, thrombocytopenia, elevated transaminases	NA	NA	PCR (+) IgG (−) IgM (−)	Fatal 8/8/18	No	[38,84]
4	53	M	08/2018	Sierra de Béjar (Salamanca)	Not confirmed	Cattle farming	Fever, chills, mouth ulcerations (not any haemorraghic oral bullae), acute leg myalgias with no bleeding symptomatology leukopenia, thrombopenia, increase of transaminases with an anicteric cholestasis, and prolongation of activated partial thromboplastin time. Also, a hemophagocytic syndrome was raised in order of the presence of hyperferritinemia (>10,000 ng/mL), hypertriglyceridemia and increase of lactate dehydrogenase (LDH).	Unspecific viriasis	NA	Retrospective (2019) PCR (+) IgG (+) IgM (+)	Survivor	No	[38,84]
5	69	M	01/06/2020	NA (Salamanca)	Yes (in the leg) 30/5/20	NA	High fever and skin rash of 24 h of evolution + epistaxis and eye redness one week later	MSF	DOX	10/06/20 PCR (+) IgG (+) IgM (+)	Survivor	No	[86]
6	53	M	29/06/2020	NA (Salamanca)	Yes	Agriculture and livestock farming	Myalgia, fever, thrombocytopenia, elevated transaminases	NA	NA	07/07/20 PCR (+) IgG (+) IgM (+)	Survivor	No	[86]
7	69	M	05/08/2020	NA (Salamanca)	Yes (in the leg)		Fever, arthralgia + digestive hemorrhage after five days	Pneumonia	AZM	12/08/20 (post- mortem)	Fatal (11/8/20)	No	[86]

Dates are formatted as: dd/mm/yy (Day/Month/Year); NA: Not available; MSF: Mediterranean spotted fever; DOX: Doxycycline; RBV: Ribavirin; AZM: Azithromycin.

**Table 4:** Seroprevalence of Crimean-Congo hemorrhagic fever virus (CCHFV) in humans in western Europe.

Country	Dates	% IgG	% Gende	Risk Factors	Method	Reference
Portugal	1980	0.8 (2/258)	NA	Living in certain areas of southern Portugal	PRN, IFA	[51]
Spain	2010–2014	0 (0/228)	NA	Hunters and tick-bite or tick-borne disease	IFA	[33]
	2017	0 (0/49)	26.5 M, 73.5 F	Family contacts and hospital workers who had attended CCHF cases	ELISA, IFA	[79]
	2017–2018	0.58–1.16 (3/516–6/516) ^1^	68.4 M, 31.6 F	Living in rural areas, contact with animals, animal husbandry, agriculture and shepherding, slaughtering, hunting, veterinary and healthcare work, tick-bite	ELISA, IFA	[83]
	2017–2018	3.0 (4/133) ^2^	60.9 M, 39.1 F	NA	ELISA, IFA	[85]

NA: not available; PRN: plaque reduction neutralization; IFA: indirect immunofluorescence assay; ELISA: Enzyme-Linked ImmunoSorbent Assay; ^1^ Asymptomatic blood donors; ^2^ Patients with acute undifferentiated febrile illness.

**Table 5:** Seroprevalence studies of Crimean Congo hemorrhagic fever virus-infected livestock in western Europe.

Country	Date	Source	% IgG	Method	Reference
Corsica (French island)	2014–2016	Cattle Goats Sheep	13.3 (231/1731) 3.1 (32/1035) 2.5 (28/1124)	ELISA	[87]
Portugal	1980	Goats	0 (0/141)	PRN, IFA	[51]
Spain	2016	Wild animals Domestic animals	58 (39/67) 33 (26/79)	ELISA	[38]
	2018	Wild animals	70 (220/314) ^1^ 79 (163/206) ^2^ 2 (2/119) ^3^	ELISA	[38]
		Domestic animals	16 (75/467) ^1^ 4 (13/309) ^2^ 7 (23/326) ^3^	ELISA	[38]

PRN: plaque reduction neutralization; IFA: indirect fluorescent antibody tests; ELISA: Enzyme-Linked Immuno-Sorbent Assay; ^1^ Zone 1: presence of CCHFV in ticks from wild or domestic animals or vegetation; ^2^ Zone 2: absence of CCHFV in ticks from wild or domestic animals or vegetation; ^3^ Zone 3: low probability of presence of *Hyalomma* spp. ticks.
